# Annealing-induced recovery of indents in thin Au(Fe) bilayer films

**DOI:** 10.3762/bjnano.7.199

**Published:** 2016-12-28

**Authors:** Anna Kosinova, Ruth Schwaiger, Leonid Klinger, Eugen Rabkin

**Affiliations:** 1 Department of Materials Science and Engineering, Technion – Israel Institute of Technology, 32000 Haifa, Israel; 2 Karlsruhe Institute of Technology, Institute for Applied Materials, PO Box 3640, 76021 Karlsruhe, Germany

**Keywords:** annealing, diffusion, dislocation loops, nanoindenation, thin films

## Abstract

We employed depth-sensing nanoindentation to produce ordered arrays of indents on the surface of 50 nm-thick Au(Fe) films deposited on sapphire substrates. The maximum depth of the indents was approximately one-half of the film thickness. The indented films were annealed at a temperature of 700 °C in a forming gas atmosphere. While the onset of solid-state dewetting was observed in the unperturbed regions of the film, no holes to the substrate were observed in the indented regions. Instead, the film annealing resulted in the formation of hillocks at the indent locations, followed by their dissipation and the formation of shallow depressions nearby after subsequent annealing treatments. This annealing-induced evolution of nanoindents was interpreted in terms of annihilation of dislocation loops generated during indentation, accompanied by the formation of nanopores at the grain boundaries and their subsequent dissolution. The application of the processes uncovered in this work show great potential for the patterning of thin films.

## Introduction

The intentional introduction of defects into bulk metallic material by plastic deformation followed by heat treatments, i.e., thermo-mechanical treatment, is a technological cornerstone of human civilization and has been used for millennia to design microstructures and properties of materials. Yet, applying this approach to thin metal films is problematic because the traditional methods of high-strain deformation, such as rolling or forging, are not applicable. Nanoindentation is widely used for characterizing the mechanical properties of nano- and micro-scale specimens. It can also be employed to produce defects of controlled shape and size, and to introduce high plastic strain in thin films. Here, we present the results of a study investigating the recovery of indentation-induced defects in Au(Fe) bilayer films. The motivation for our work is two-fold: (i) to explore the concept of thermo-mechanical treatment of thin films by combining localized plastic strain introduced by nanoindentation with subsequent annealing, and (ii) to understand the effect of nanoindentation-induced localized plastic deformation on the thermal stability and solid-state dewetting of thin films. In what follows, we will give a short overview of what is known about the recovery of indents in thin metal films at elevated temperatures and about solid-state dewetting of thin metal films.

To the best of our knowledge, very few publications report studies of the thermo-mechanical processing of thin metal films by combining nanoindentation and annealing. Lee et al. [1–2] investigated the microstructural changes and phase transformations induced by nanoindentation and annealing in Ni [1] and Ag thin films [2] on Si substrates. They revealed that the distortion of the crystalline structure induced by indentation enhances the diffusivity of metal atoms and prompts the formation of nickel and silver silicide phases. Similar enhancement of diffusivity and the formation of a eutectic phase were observed in the indented region of a Au thin film on Si (with a Cr interlayer) [3]. Also, the material pileup around the indents in the as-deposited film fully recovered and disappeared after annealing in the temperature range of 250–450 °C [3]. It should be noted that in all three works [1–3] the maximum indentation depth was larger than the metal film thickness, which resulted in significant modification of the Si substrate. Still, the microscopic mechanisms responsible for redistribution of matter in the indented region and the microstructural changes caused by annealing remained poorly understood.

Annealing-induced shape recovery of indents produced by nanoindentation is well-documented for thin nickel–titanium films; yet, in this case the recovery is related to diffusionless martensite–austenite transformation [4]. Thermally activated strain recovery has been reported for thin Al and Au films [5]. It has been suggested that plastic deformation recovery is restricted to small grain sizes and driven by inhomogeneous residual internal stresses. The above short overview shows that the understanding of the processes occurring in deformed thin metal films upon annealing is quite poor compared to the great wealth of data on recovery and recrystallization of deformed bulk metals [6].

The introduction of defects into thin metal films can significantly affect their thermal and mechanical stability. For example, solid-state dewetting of thin metal films is a well-known phenomenon, which results in temperature-induced morphology changes, exposure of the substrate, and transformation of a film into an array of individual particles. This process is driven by the reduction of the total surface and interfacial energy of the system. Since a wide range of applications of thin metal films in the microelectronics requires featureless and defect-free surfaces, the knowledge of the effect of localized plastic strain on dewetting is of high practical importance. Surface and interface defects, such as grooves at grain boundaries and triple junctions [7–8], voids at the interface between the film and the substrate [9], and localized impurities on the substrate or on the film surface can serve as nucleation sites for the onset of solid-state dewetting upon heating. Localized plastic deformation by indents on the film surface is expected to play a two-fold role in dewetting. On the one hand, the indents could serve as additional nucleation sites for the holes and, thus, accelerate the dewetting process. On the other hand, they could add fast diffusion paths in the near-surface region and, thus, accelerate the healing processes in the material controlled by capillary forces [10–11] or the interaction of defects [12]. The healing capability of defects was recently demonstrated for Au [13] and Fe [14] nanoparticles. It was shown that the controlled injection of dislocations into single-crystalline faceted metal nanoparticles results in their much faster equilibration, i.e., achieving the equilibrium crystal shape, during subsequent annealing as compared to their defect-free counterparts [13–14].

Recently, Amram and Rabkin [15] studied the role of Fe underlayers in the growth of quasi-single-crystalline heteroepitaxial Au thin films on sapphire. The Au layer deposited on Fe exhibited a high degree of in-plane ordering with a large grain size. In the Au(Fe)-sapphire system, the thin Fe underlayer reduces the lattice mismatch between Au and sapphire and increases the adhesion energy, which lowers the concentration of structural defects in the film and improves its thermal stability. The Au layer exhibited a strong [111] out-of-plane texture and two twinning-related in-plane orientations related by a 60° rotation around the surface normal. The Fe underlayer exhibited a strong [110] out-of-plane texture with three in-plane orientations. The morphological stability and initial stages of dewetting of quasi-single-crystalline Au(Fe) thin films has been described in detail in [16]. Thus, this system represents a convenient model object for the studies of the effect of indentation-induced surface defects on the film behavior at elevated temperatures. This is because these films exhibit only one type of grain boundaries (Σ3 twin boundary, where Σ is a reciprocal density of coincident sites) and are intrinsically stable against dewetting. The interdiffusion coefficients in the Au(Fe) system have been determined by Iijima and Yamazaki [17]. At the temperature of 700 °C employed in the present study, the full intermixing of a 50 nm thick bilayer takes less than 1 s, and, therefore, in what follows we will refer to the Au(Fe) bilayers as a single-phase face-centered cubic dilute solid solution of Fe in Au, or Au(Fe) thin films.

The goals of the present work were to determine the thermal stability of Au(Fe) films containing indentation-induced surface defects, and to establish the mechanisms of indent recovery. The latter goal should be considered in a wider framework of thermo-mechanical processing of thin metal films. The localized defects in the film were introduced by load-controlled nanoindentation. We employed atomic force microscopy (AFM) for studying the surface morphology evolution upon annealing; special attention was paid to the healing processes in the indented area and the dewetting process in the unperturbed film.

## Results

**Figure 1:**
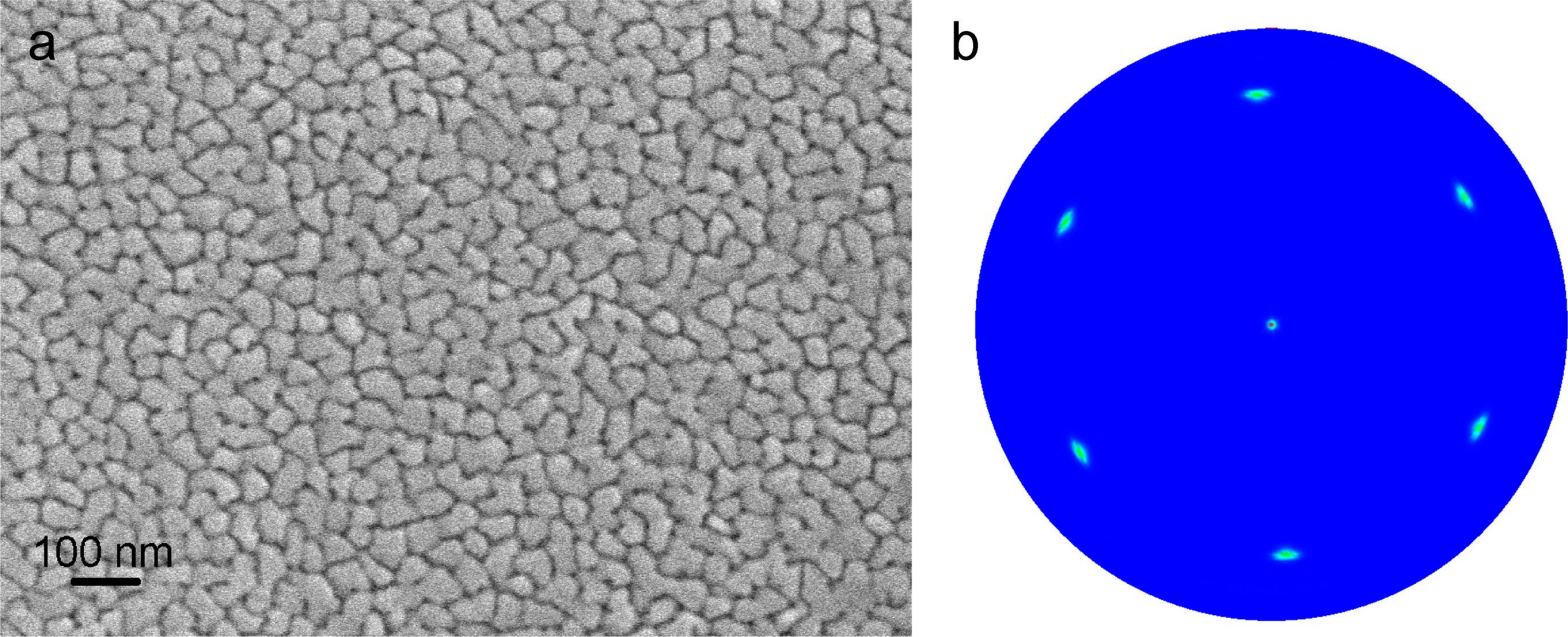
The microstructure of the as-deposited Au(Fe) film. (a) High-resolution scanning electron microscopy (SEM) micrograph of the Au(Fe) film, which contains grains of 40 to 50 nm in diameter; (b) XRD pole figure of the Au {111} reflections from the as-deposited Au(Fe) film. Two orientation variants are distinguishable, rotated by 60° with respect to each other around the [111] surface normal.

The microstructure of the ultrathin (5–20 nm) Au(Fe) films has been described in detail in [15]. It was shown that the deposition of a Au film on a thin Fe underlayer resulted in a high-quality quasi-single-crystalline Au films with large grains having diameters of several micrometers. In the case of the thicker films studied in the present work (the total thickness is 50 nm), the average grain size was comparable to the film thickness (Figure 1a). Triple junctions are clearly observed between the majority of grains, which is typical of a polycrystalline film. The presence of triple junctions in the film is related to the deviations from the ideal Σ3 misorientation between the grains, as also confirmed by an angular spread of the {111} reflections in the X-ray diffraction (XRD) pole figure shown in Figure 1b. Indeed, thin films with the perfect <111> texture, in which all grain boundaries are of the exact <111>Σ3 type, so-called mazed bicrystalline films [18–19], exhibit a special type of microstructure without triple junctions. The electron backscatter diffraction data obtained on the as-deposited film (not shown here) confirmed that only the near-<111>Σ3 tilt grain boundaries, and low-angle grain boundaries were present in the film.

**Figure 2:**
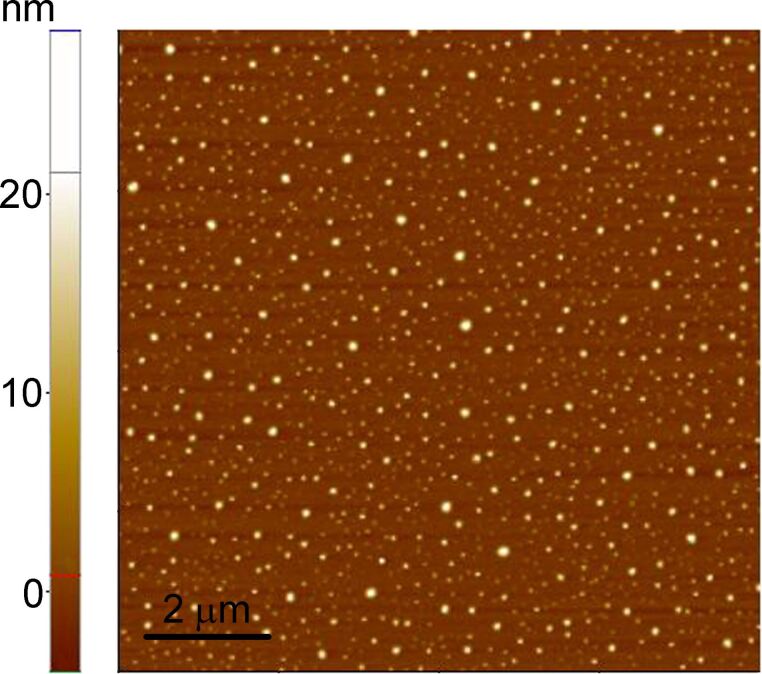
AFM micrograph of the surface of the as-deposited Au(Fe) film, four months after deposition.

The atomic force microscopy (AFM) image of the Au(Fe) film surface (Figure 2) was taken four months after the film deposition. Hillocks with a height up to 25 nm were present on the film surface, which could be the result of slow stress relaxation in the as-deposited Au(Fe) bilayers at room temperature. After the first annealing for 10 min, the hillocks disappeared.

**Figure 3:**
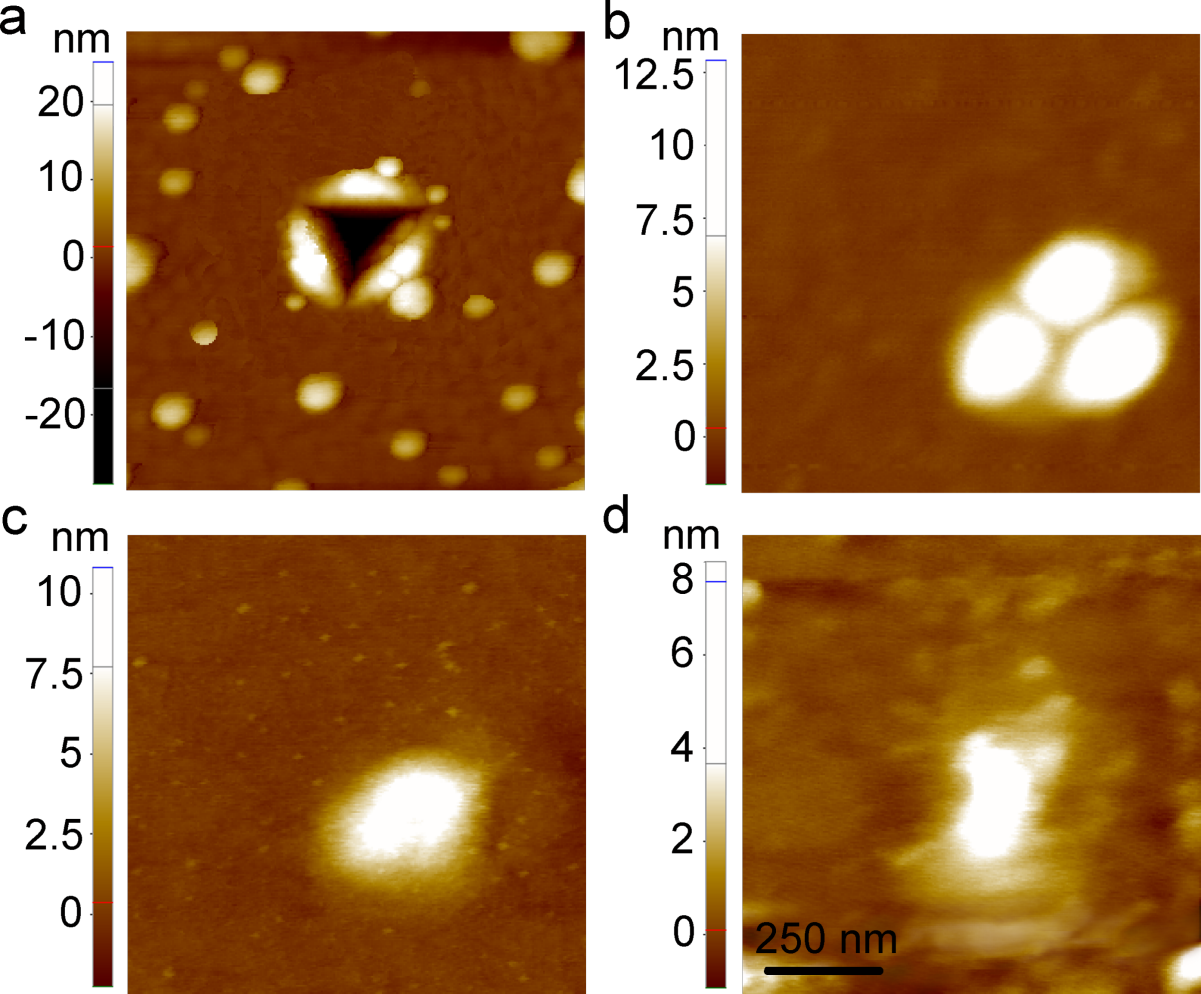
AFM topography images of one and the same imprint produced at *P* = 0.2 mN (a) after indentation, after annealing for (b) 10 min, (c) 20 min, (d) 290 min. Cumulative annealing times are given.

AFM line profiles were used to determine the indent depth before and after annealing. The average depths of the as-produced indents were 18 and 25 nm for the maximum indentation loads of 0.1 and 0.2 mN, respectively, which is less than half the film thickness. Since a three-sided pyramidal, Berkovich tip was used for the indentation, the initial indent shape was triangular (Figure 3a). Setting the level of the unperturbed ﬁlm to zero, we calculated the amount of piled-up (positive) and displaced (negative) material by integrating all negative and positive pixels in the indented region. The volumes of piled-up and displaced material were approximately balanced.

**Figure 4:**
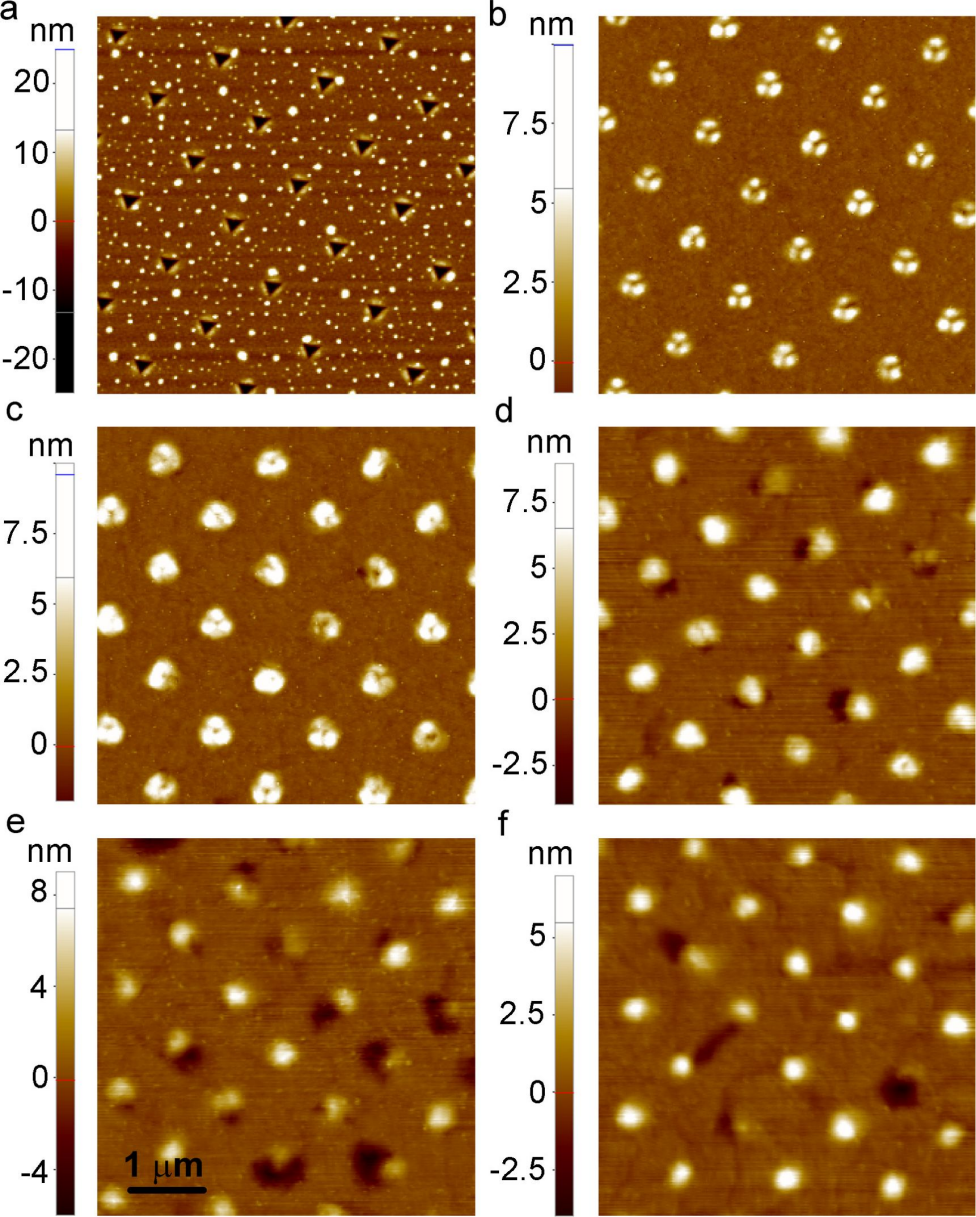
AFM topography images of nanoimprints produced at *P* = 0.2 mN (a) after indentation, after annealing for (b) 10 min, (c) 20 min, (d) 30 min, (e) 110 min, (f) 290 min. Cumulative annealing times are given.

**Figure 5:**
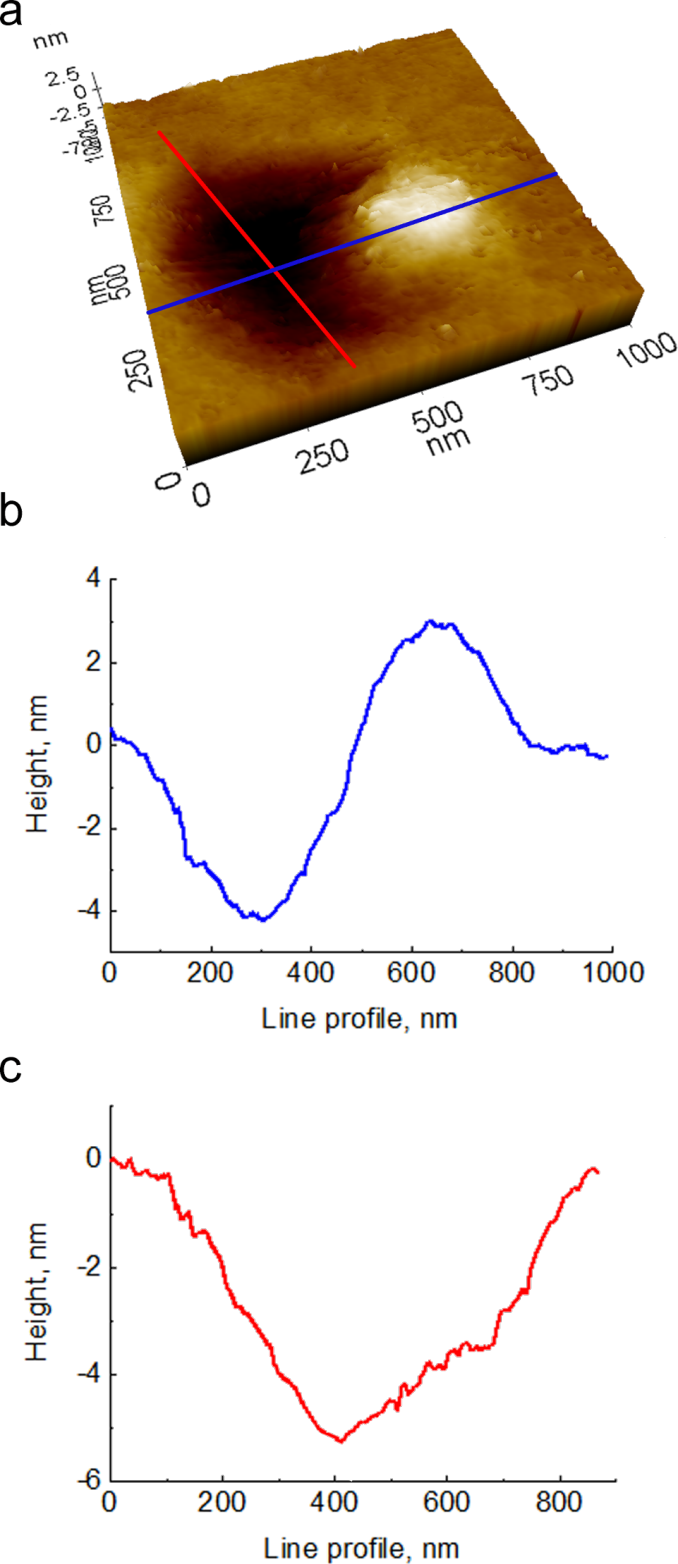
A three-dimensional AFM image of the indent after annealing for (a) 290 min and AFM profiles taken along the (b) blue and (c) red lines.

Thermally induced morphology evolution of the indents produced at the maximum load of *P* = 0.2 mN is shown in Figure 3 for one and the same indent, and in Figure 4 for random locations within the indented area. Upon heating, the healing process of the indents was thermally initiated. After annealing for 10 min, the piled-up material transformed into three-lobe hillocks (Figure 3b and Figure 4b), which resulted in a decrease of the maximum indent depth and in the lateral expansion of the indent. After the next heat treatment, the three lobes tend to merge into a single hillock (Figure 3c and Figure 4c). Annealing for 30 min results in the formation of near-hillock surface depressions accompanied by a slight reduction of the hillock height (Figure 4d). The depressions were observed near approximately 40% of all hillocks, which may have been caused by a local variation of the kinetic parameters or microstructure inhomogeneities. Interestingly, during subsequent annealing treatments, the hillocks with and without a nearby depression behaved differently. The height of the hillocks without a nearby depression stabilized at a value of 6–8 nm after the hillocks had been fully formed, and did not change upon subsequent anneals. The hillock-depression couples did change their morphology, with the depression becoming deeper consuming the nearby hillock. The latter process resulted in a shallow cavity adjacent to the disappearing hillock (Figure 4e and Figure 4f). Figure 5 shows the typical morphology of an indent transformed into a hillock-depression couple after prolonged annealing (290 min). The depth of the depression is about 5 nm, while the hillock height is reduced to 3 nm. It is worth noting that the depression has an elongated shape with gently sloping edges and a root exhibiting an abrupt change of the surface slope (Figure 5c). This fact testifies to a significant contribution of grain boundaries to the depression formation. Indeed, an abrupt change of the surface slope is an essential attribute of the grain boundary groove, indicating a balance of surface and grain boundary energies at the triple line [7,20]. Our EBSD observations have demonstrated that significant grain growth and texture sharpening had occurred in the film upon annealing, so that either a single grain boundary or no grain boundary at all were present in the immediate vicinity of the indents. We believe that the depressions were formed only in the cases when a grain boundary was available near the hillock.

The indents produced at *P* = 0.1 mN behaved in a similar manner. For these smaller indents, the formation of a single hillock followed by the appearance of a depression occurred slightly faster than in the case of indents produced at *P* = 0.2 mN. After annealing for 10 min, most of the indents were transformed into single hillocks, while some indents had a slightly distinguishable three-lobe shape. The depth of the shallow depressions did not exceed 5 nm after the last heat treatment.

**Figure 6:**
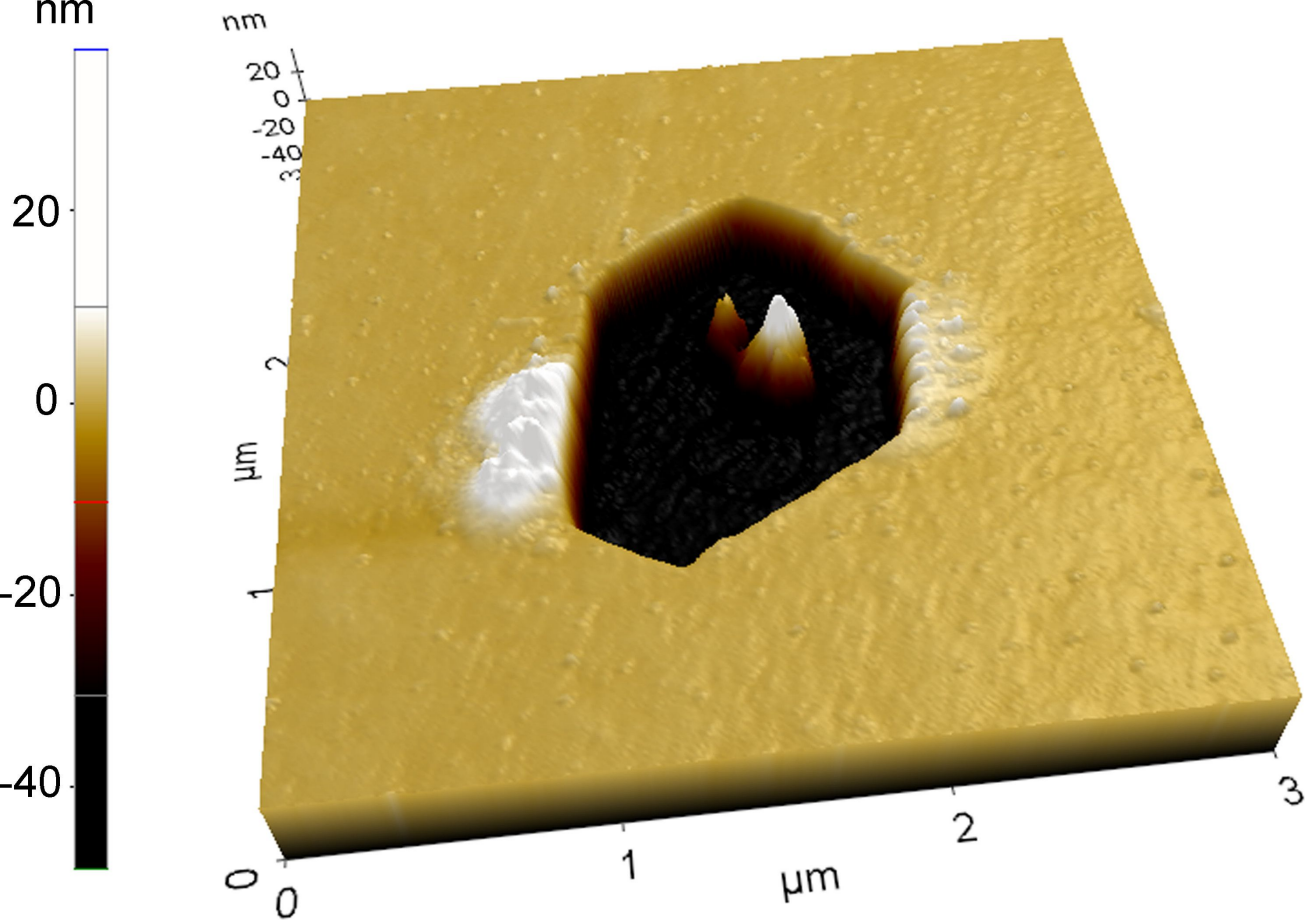
Three-dimensional AFM image of the hole in the Au(Fe) film far from the indented regions after annealing for 20 min.

We also examined the film morphology far from the indented area. The onset of dewetting was observed right after the first annealing. The holes were randomly distributed and exhibited hexagonal symmetry. All holes nucleated at once, i.e., no new nucleation events were observed during subsequent anneals, and their density was about 5 × 10^8^ m^−2^. A typical hole formed during solid-state dewetting of the film is shown in Figure 6. The hexagonal shape of the holes tends to transform into a triangular one over time, as it was observed for a Au(Fe) film with lower thickness [16]. This fact is attributed to the faster surface diffusion along the disappearing facets. In contrast to the holes observed in [16], the holes observed in this study did not exhibit elevated rims. Yet, most of the holes were surrounded by a ring of small bumps. The volume of the material in the bumps is much smaller compared to the material removed from the hole. Hillocks as observed in the as-deposited film were not observed. This raises a question about the relocation of material consumed by the hole. It is reasonable to assume that the material is redistributed along the film–substrate interface, similar to what has been observed in Ni thin films with the mazed bicrystal microstructure [21]. This type of dewetting greatly differs from the classical one, implying the accumulation of the material in the rims of the holes [22]. The central feature inside the hole indicates its nucleation site. Such features were observed in many studies of the solid-state dewetting, yet the mechanism of their formation is still unknown [22].

## Discussion

In this study, we found that thermal treatment results in recovery of indents produced at the applied loads of 0.1 and 0.2 mN, with depths reaching up to half of the film thickness. It is interesting that no holes nucleated in the indented regions, whereas the dewetting process had started in the pristine, unperturbed regions of the film. This means that shallow indents, which do not reach the substrate, do not promote dewetting. The healing process does not lead to an entirely flat metal surface, but results in a hillock-depression morphology instead. The apparent stability of these hillock-depression couples can be explained by their low surface curvature, and, hence, low driving force for surface diffusion, which controls the flattening process [11]. Yet, the intermittent stage of relaxation, in which the hillock is formed at the site of the indent, is very counterintuitive and apparently defies the condition of mass conservation. In what follows, we will discuss the possible mechanisms of indent→hillock→depression transformations. This chain of relaxation processes is a result of a complex, synergetic interplay of different types of defects, including dislocation loops, grain boundaries, and pores, and diffusion paths, such as the surface, grain boundaries, and the film–substrate interface, which were either present in the as-deposited film or produced during indentation.

**Figure 7:**
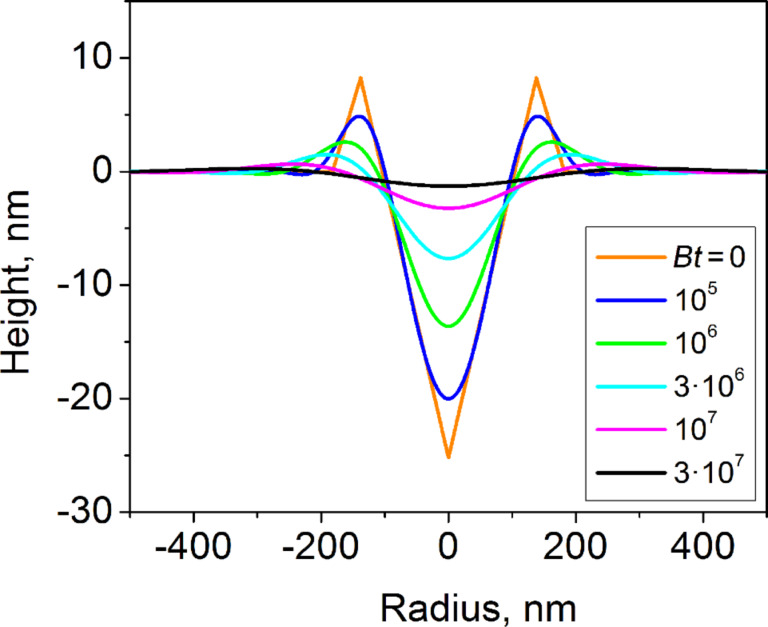
The simulated surface topography of the indent evolving by a surface diffusion mechanism, for different annealing times. *B* and *t* are the Mullins coefficient and the annealing time, respectively (see Appendix).

Let us first demonstrate that surface diffusion alone cannot explain the transformation of a volume-conserving surface indent into a hillock. Such a transformation could occur because Mullins’ solution of the governing equation describing surface diffusion-controlled morphology evolution of a surface with a singular perturbation exhibits secondary and higher-order extrema in the surface profile [7]. Thus, the indents of certain morphologies can evolve into a central hillock surrounded by a shallow, but wide depression. We considered a conical, axisymmetrical indent with volume-conserving pileups and area function corresponding to a Berkovich indenter tip (see Appendix for details). We employed a small-slope approximation [7] and calculated the evolution of the topography of the indented region by a surface diffusion mechanism (Figure 7). For short annealing times, the bottom of the indent and the tops of the pileups undergo fast smoothing, while the walls of the indent remain almost straight. For long annealing times, the topography evolves into a minor central depression surrounded by a slightly elevated ring. At this stage, the evolution of the surface slows down drastically because of the decrease in surface curvature. Thus, these simulations demonstrate that surface diffusion alone cannot lead to the transformation of an indent into a hillock.

It is important to note that the pre-existing hillocks on the film surface (Figure 2) do not play any significant role in annealing-induced evolution of the film topography. We performed several control indentation-annealing experiments with the as-deposited Au(Fe) film free of surface hillocks. This film exhibited the same phenomenon of indent recovery accompanied by the formation of characteristic hillock-depression couples.

**Figure 8:**
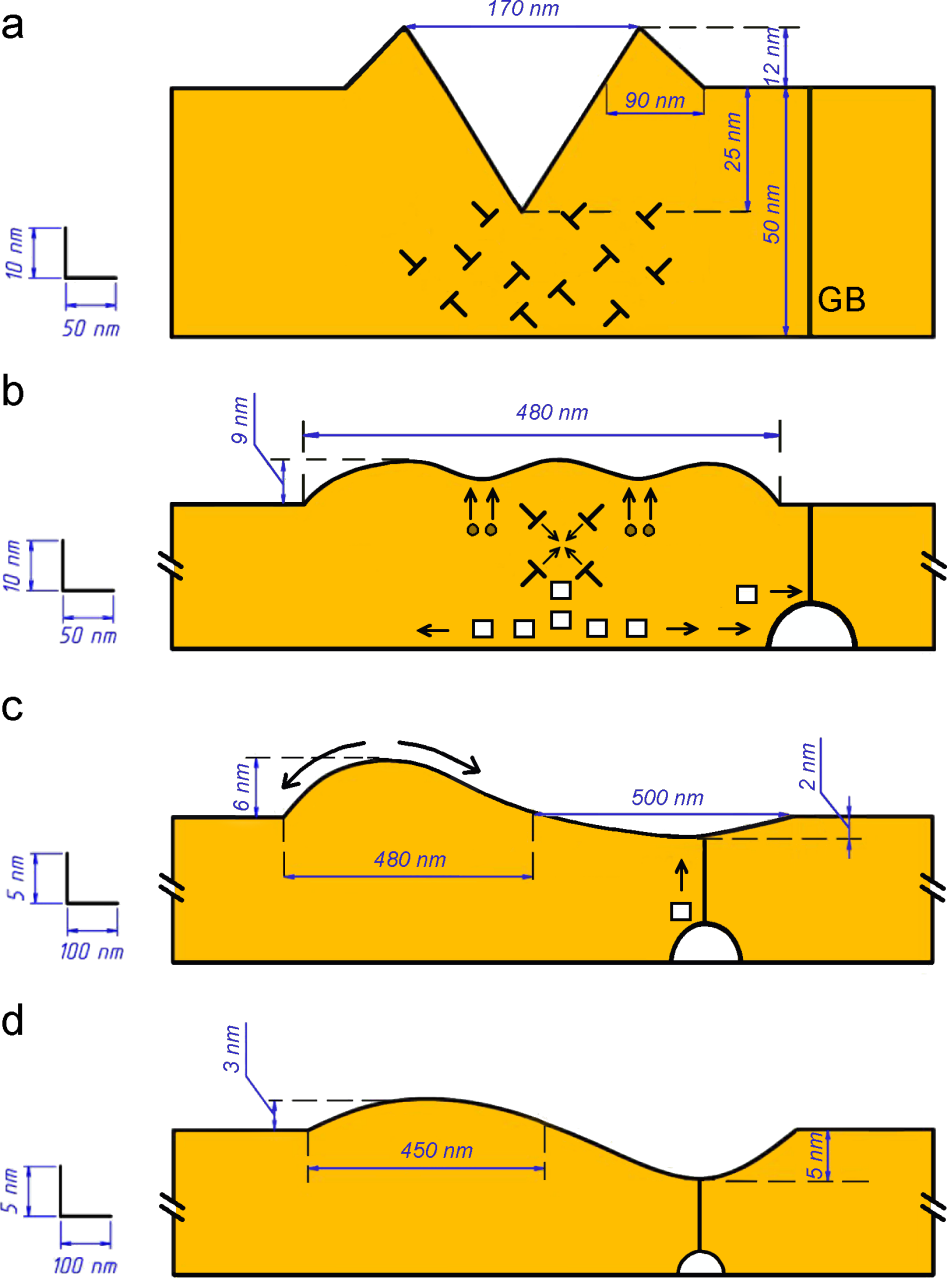
Schematic illustration of indent evolution with increasing annealing time: (a) before annealing, (b) 20 min, (c) 50 min, (d) 290 min. Various topography features are shown in-scale (as indicated by the scale bars on the left hand side of the figure), and their dimensions were obtained by averaging of AFM topography data for several indents. It should be noted that the arrangement of the dislocations shown does not represent the crystallography of the sample, but rather symbolizes the high dislocation density accounting for mass conservation and that is typical of indentation. The envisioned process should occur for all crystal orientations.

**Figure 9:**
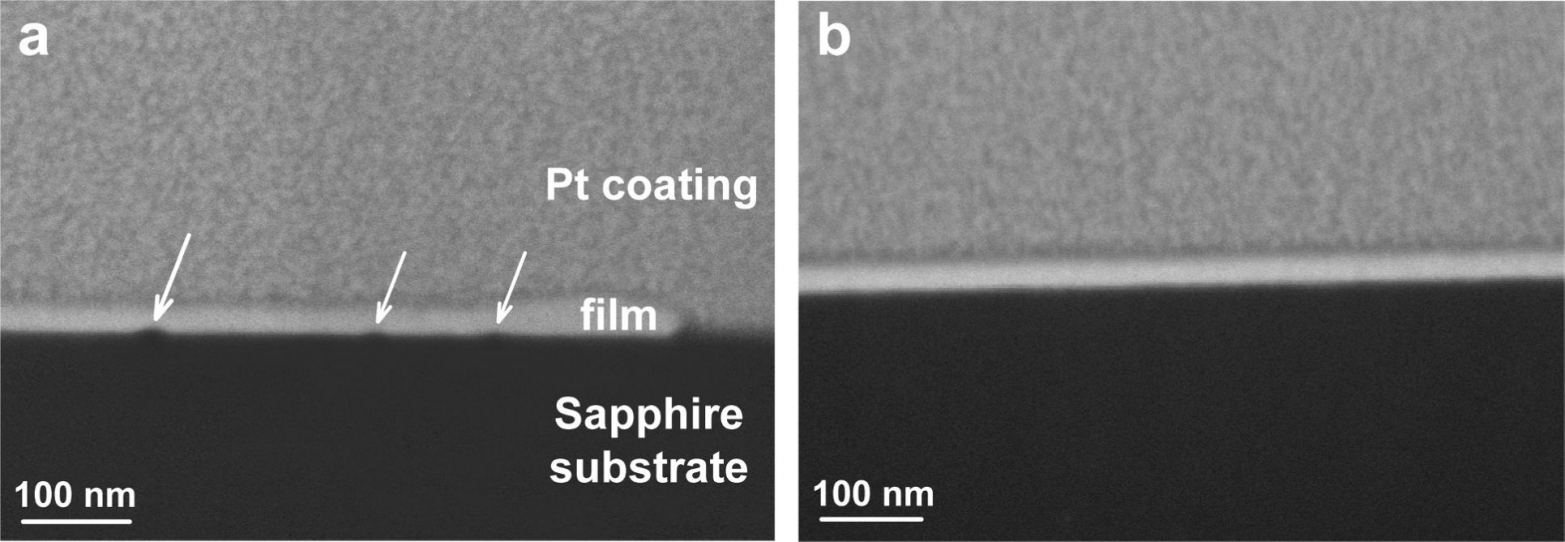
(a) Cross-sectional SEM image from the region indented at a high load (*P* = 1 mN), and (b) of the unperturbed region of the film. The images were taken under the tilt angle of 35°. One can see the indent that reached to the substrate (on the right side of the image (a), where the Pt coating covers the substrate directly) and the pores at the film–substrate interface (indicated by arrows). The annealing time was 10 min.

We believe that prismatic dislocation loops produced during indentation are responsible for the upward relaxation of the indent. We will assume that intensive plastic flow of the material around and underneath the indenter during indentation results both in the formation of material pileups, and of dislocation loops partly compensating for the displaced material in the indent (see Figure 8a). Some hints about the geometry of the plastic flow during a nanoindentation test consistent with the schematic model presented in Figure 8 can be obtained from the nanoindentation experiments on metallic multilayers [23]. Also, the nature of prismatic dislocation loops in the indented region has been recently discussed by Gagel et al. [24]. In Figure 8, we present the schematic drawings of the film topography after different annealing times (based on experimentally measured topography profiles averaged for several indents), together with the schematic presentation of the corresponding changes of the material microstructure. Upon annealing, the dislocation loops will tend to annihilate, both under the action of their own line tension and the residual stresses in the indented region. These residual stresses arise due to strong adhesion between the film and the substrate as well as the plastic strain associated with the indentation. The annihilation of the dislocation loops, which proceeds by a dislocation climb mechanism, leads to the elevation of the middle part of the indent and the formation of a hillock, with the surface diffusion causing the edges to smoothen and the affected surface region to widen (Figure 8b). Since the annihilation of dislocation loops is non-conservative, it generates a flux of excess vacancies. The vacancies can reach the film-substrate interface and the nearby grain boundary, which both can serve as vacancy sinks. The annihilation of vacancies at the film-substrate interface leads to the slight decrease of the film thickness [21], whereas annihilation of vacancies at the grain boundaries helps to relax compressive stresses in the film formed during heating due to the mismatch of thermal expansion coefficients between the film and the substrate. The rim-less shape of the dewetting holes observed far from the indented region (see Figure 6) confirms that the film-substrate interface is indeed a potent sink and source of vacancies; the vacancies that originated at the interface are consumed by the expanding hole. Some fraction of the vacancy flux generated by annihilating dislocation loops may lead to the nucleation of a pore, with the triple line where the grain boundary emerges at the film–substrate interface being a preferable site for this nucleation (see Figure 8b). Such pores were observed in our studies of the large indents, which reached to the substrate, in the same thin film, see Figure 9 [25], where the pores at the film–substrate interface with a diameter of 10–20 nm can be clearly seen. In the present case of low-load indents, the pores were probably too small to be detected with the aid of the employed sample preparation method. Once the pore is formed, it begins to drain the film material from the triple line at the film surface, leading to the formation of a near-hillock surface depression, see Figure 8c and Figure 8d. In the meantime, the hillock continues to dissipate by surface diffusion. Part of the material rejected by the shrinking hillock arrives at the grain boundary and contributes to the dissipation of the pore. At the end, the film should return to its original, planar state, slightly disturbed by the grain boundary grooves. According to this scenario, an apparent violation of mass conservation in Figure 8b and Figure 8c is compensated for by the grain boundary pore, by a slight decrease of the local film thickness, and by the relaxation of the compressive stresses in the film. It should be noted that a similar apparent violation of the mass conservation condition was observed during nanoindentation studies of thin Ni films, and it was also attributed to the formation of sub-surface interface and grain boundary pores or cracks [26]. A simple, mass-conservation based estimate of the dislocation density, ρ, which is required to produce the hillock with typical dimensions shown in Figure 8b yielded ρ ≈ 10^16^ m^−2^, which is close to the maximum dislocation density in heavily deformed metals [27].

According to the proposed qualitative model, the annealing-induced evolution of indented metal is correlated to the following changes of the material microstructure in the indented region: (i) indentation-induced plastic flow and formation of prismatic dislocation loops, (ii) annihilation of the loops, formation of the hillock, and generation of excess vacancies and the grain boundary pore, (iii) dissipation of the hillock and the grain boundary pore by surface and grain boundary diffusion mechanisms. This morphology transformation is controlled by irreversible processes, such as dislocations climb, surface diffusion, and grain boundary diffusion. It is interesting that reversible formation/annihilation of hillocks at the indented sites upon heating/cooling was demonstrated in bulk and thin film samples of shape memory NiTi alloy [28]. This was achieved by planarizing the as-produced indents by mechanical polishing. The mechanism of the hillock formation upon heating is in that case related to the martensite–austenite phase transformation, which is affected by residual stress and very different from the irreversible mechanisms discussed in the present study.

## Conclusion

From the results of the present study, the following conclusions can be drawn:

1. Thin polycrystalline Au(Fe) films on basal-plane oriented sapphire substrates were produced by electron beam deposition. The films exhibited a strong [111] texture and all grain boundaries were either of the near-Σ3<111> tilt, or of the low-angle type.
2. The shallow indents reaching up to one half of the film thickness, produced by depth-sensing nanoindentation did not affect the thermal stability of the film against the solid-state dewetting.
3. Upon heat treatments at a temperature of 700 °C in a forming gas atmosphere, the indents evolve into hillocks, in some cases followed by the formation of a near-hillock surface depression. For the long annealing times, the depression becomes deeper, while the hillock is dissipated.
4. We demonstrated quantitatively that the hillock formation at the indented site cannot be explained by surface diffusion alone. We proposed a qualitative scenario in which the hillock formation is related to the climb-controlled annihilation of prismatic dislocation loops formed during nanoindentation. The annihilation generates excess vacancies, which condense in the pore at the triple line between the grain boundary and the film-substrate interface. Later, the pore absorbs the material from the film surface by grain boundary diffusion, leading to the development of the near-hillock depression.

The phenomenon observed in this work can be employed for the design of surface topography of thin metal films. While patterning with the aid of nanoindentation imprints is quite obvious, we demonstrated that a thermo-mechanical treatment of the film, i.e., nanoindentation followed by annealing, can result in the formation of ordered arrays of hillocks. While the parameters of the naturally formed hillocks can hardly be controlled, the size, spacing, and arrangement of the hillocks produced by the suggested thermo-mechanical treatment can be fine-tuned by nanoindentation parameters, such as load and indent spacing, and annealing duration and temperature.

## Experimental

Au(Fe) thin films (48 nm thick Au film on a 2 nm thick Fe underlayer) were deposited on *c*-plane sapphire (α-Al_2_O_3_) substrates at room temperature using electron beam deposition. The deposition took place in a VST e-beam evaporator with a base pressure of 5 × 10^−7^ Torr (6.7 × 10^−7^ mbar). Deposition rates were 0.2–0.3 Å·s^−1^ for Fe and 0.7 Å·s^−1^ for Au.

After the deposition, the films were patterned by square arrays of nanoimprints using a G200 DCM II nanoindenter (Agilent/Keysight Technologies) equipped with the Express Test and NanoVision options. Five big indents were used as markers in order to identify the locations of the different arrays with an optical microscope. For load-controlled nanoindentation tests, a three-sided diamond pyramidal Berkovich tip of approximately 20 nm radius of curvature at the apex was used. The arrays of nanoimprints (array size 100 × 100 µm) were produced on the film surface at applied loads of 0.1 and 0.2 mN, and contained 100 × 100 nanoimprints, so that the distance between the imprints was 1 µm. For both loading conditions, the penetration depth was significantly lower than the film thickness.

The indented samples were annealed in a quartz tube resistance furnace under forming gas flow (Ar + 10% H_2_, 99.999% pure) at a temperature of 700 °C for the consecutive time intervals of 10, 10, 10, 20, 60 and 180 min. The samples were placed in a quartz boat and introduced to the hot zone of the furnace by a mechanical manipulator. The heating time of the boat containing the sample from room temperature to 700 °C was 3 min, followed by an isothermal annealing for the required time. Quenching was achieved by rapid withdrawal of the boat with the sample from the hot zone.

After each treatment, the samples were characterized by atomic force microscopy (AFM; Park Systems XE-70) in intermittent-contact mode, using NSG30 Si probes supplied by NT-MDT with an average nominal value of resonant frequency of 270 kHz, and an average nominal radius of curvature at the apex of <25 nm.

The microstructure of the films was characterized by high-resolution scanning electron microscopy (HRSEM; Zeiss Ultra Plus), and by X-ray diffraction (XRD; Rigaku SmartLab) using Cu Kα radiation in a parallel beam configuration. HRSEM micrographs were taken using a secondary electron detector, at an acceleration voltage of 3 kV. Pole figures were measured using slit collimation. Precise alignment of the samples was performed using rocking curves (ω scans) on the Au (111) reflection. Cross-section samples were prepared in a dual-beam focused ion beam microscope (FIB; FEI Strata 400-S).

## Appendix

### Topography evolution of an axisymmetrical indent by surface diffusion (small slope approximation)

To check whether surface diffusion alone can lead to indent transformation into a hillock, we simulated topography evolution of an axisymmetrical conical indent in a small-slope approximation.

The axial surface is described by the topography profile *Y* = *Y*(*r,t*), where *r* is the distance from the central axis and *t* is the annealing time. We assume that ∂*Y*/∂*r* << 1, so that the small-slope approximation can be used. The chemical potential of atoms on the surface, µ, is proportional to a local curvature of the surface µ = Ωγκ, where the curvature, κ, is given by

[1]
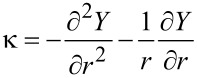


and Ω, γ are the atomic volume and the surface energy, respectively. The diffusion flux, *j*, is proportional to the gradient of the chemical potential

[2]
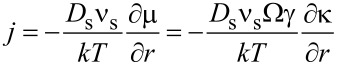


The accumulation of atoms on the surface is proportional to the divergence of the diffusion flux. Thus,

[3]
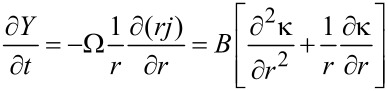


where


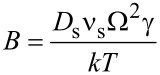


is the Mullins coefficient. *D*_s_ and *ν*_s_ are the surface self-diffusion coefficient and the number of mobile atoms per unit area of the surface, respectively, and *kT* has its usual thermodynamic meaning. Equation 1–3 represent full analogs of Mullins equations for a cylindrical surface.

### Numerical scheme

For the numerical solution of Equation 1–3, we introduce the circles *r_n_* = *n*Δ and *Y_n_* = *Y*(*r_n_*), 0 ≤ *n* ≤ *N*. We will approximate the shape of the surface between the circles *r_n_* by a cone. The chemical potential of atoms on the surface for *r* = *r_n_* is determined as


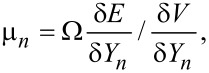



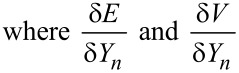


are the variations of the surface energy and the cone volume, respectively, with the displacement of the *y*-coordinate of the *n*th circle. Direct calculation yields the following expressions:

[4]
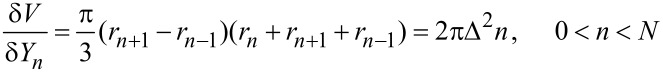


[5]
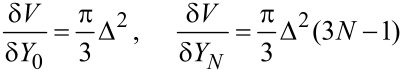


[6]
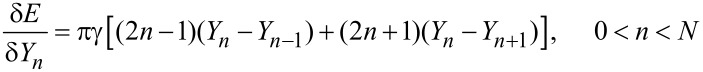


[7]
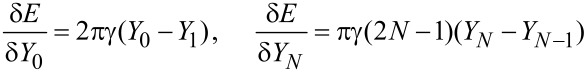


Combining the Equations 4–7 yields µ*_n_* = Ωγκ*_n_*, where

[8]
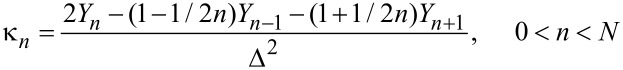


[9]
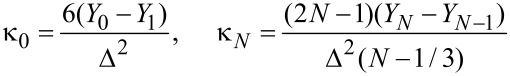


The total atomic flux between the circles *n* and *n* + 1 is

[10]
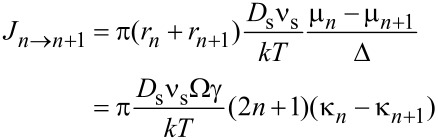


Final kinetic equations follow from the condition of mass balance:

[11]
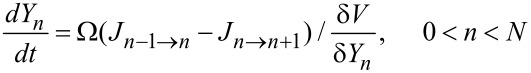


[12]
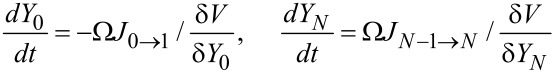


Substituting Equations 4, 5 and 10 in Equations 11, 12 yields:

[13]
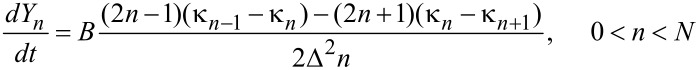


[14]
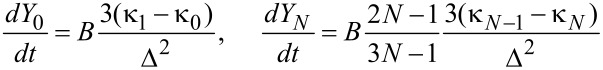


The set of the ordinary differential equations (Equations 8, 9 and Equations 13, 14) was solved employing the standard Euler method with the parameter *Bdt*/Δ^4^ < 0.05. The results of the simulations are shown in Figure 7.

## References

[1] Lee W-S, Chen T-H, Lin C-F, Chen J-M (2010). Appl Phys A.

[2] Lee W-S, Chen T-H, Lin C-F, Wu C-L (2011). Mater Trans.

[3] Lee W-S, Fong F-J (2008). Mater Sci Eng, A.

[4] Shaw G A, Stone D S, Johnson A D, Ellis A B, Crone W C (2003). Appl Phys Lett.

[5] Rajagopalan J, Han J H, Taher M, Saif A (2007). Science.

[6] Humphreys F J, Hatherly M (2004). Recrystallization and related annealing phenomena.

[7] Mullins W W (1957). J Appl Phys.

[8] Génin F Y, Mullins W W, Wynblatt P (1992). Acta Metall Mater.

[9] Shaffir E, Riess I, Kaplan W D (2009). Acta Mater.

[10] Packard C E, Schroers J, Schuh C A (2009). Scr Mater.

[11] Mullins W W (1959). J Appl Phys.

[12] Xu G Q, Demkowicz M J (2013). Phys Rev Lett.

[13] Kovalenko O, Rabkin E (2015). Scr Mater.

[14] Kovalenko O, Chikli F O, Rabkin E (2016). Scr Mater.

[15] Amram D, Rabkin E (2013). Acta Mater.

[16] Amram D, Klinger L, Rabkin E (2012). Acta Mater.

[17] Iijima Y, Yamazaki Y (2005). Defect Diffus Forum.

[18] Radetic T, Ophus C, Omsted D L, Asta M, Dahmen U (2012). Acta Mater.

[19] Dehm G, Inkson B J, Wagner T (2002). Acta Mater.

[20] Rabkin E, Amouyal Y, Klinger L (2004). Acta Mater.

[21] Amram D, Klinger L, Gazit N, Gluska H, Rabkin E (2014). Acta Mater.

[22] Thompson C V (2012). Annu Rev Mater Res.

[23] Zhang G P, Liu Y, Wang W, Tan J (2006). Appl Phys Lett.

[24] Gagel J, Weygand D, Gumbsch P (2016). Acta Mater.

[R25] 25Kosinova, A.; Schwaiger, R.; Kraft, O.; Rabkin, E. to be submitted to *Acta Materialia*.

[26] Rabkin E, Deuschle J K, Baretzky B (2010). Acta Mater.

[27] Callister W D, Rethwisch D G (2012). Fundamentals of Materials Science and Engineering: an Integrated Approach.

[28] Zhang Y, Cheng Y-T, Grummon D S (2006). Appl Phys Lett.

